# Near-infrared to visible and ultraviolet upconversion in TiO_2_ thin films modified with Er and Yb

**DOI:** 10.1039/d2ra08305a

**Published:** 2023-03-07

**Authors:** Anna Śliz, Marta Radecka, Piotr Jeleń, Dominik Dorosz, Katarzyna Zakrzewska

**Affiliations:** a Faculty of Materials Science and Ceramics, AGH University of Science and Technology al. A. Mickiewicza 30 30-059 Kraków Poland annakot@agh.edu.pl; b Faculty of Computer Science, Electronics and Telecommunications, AGH University of Science and Technology al. A. Mickiewicza 30 30-059 Kraków Poland

## Abstract

Upconversion as a modification strategy to enhance the utilization of sunlight in titanium dioxide photoanodes with an internal upconverter was investigated. TiO_2_ thin films containing an Er activator and Yb sensitizer were deposited in the magnetron sputtering process on conducting glass, amorphous silica, and silicon. Scanning electron microscopy, energy dispersive spectroscopy, grazing incidence X-ray diffraction, and X-ray absorption spectroscopy allowed assessment of the thin film composition, structure, and microstructure. Optical and photoluminescence properties were measured by means of spectrophotometry and spectrofluorometry. Changing the content of Er^3+^ (1, 2, 10 at%) and Yb^3+^ (1, 10 at%) ions allowed us to achieve thin film upconverters with a crystallized and amorphous host. Upon 980 nm laser excitation Er^3+^ exhibits upconversion with the main emission in green (^2^H_11/2_ → ^4^I_15/2_, *λ*_em_ ≈ 525 nm) and weak emission in red (^4^F_9/2_ → ^4^I_15/2_, *λ*_em_ ≈ 660 nm). For a thin film with a higher ytterbium content (10 at%) a significant increase in red emission and upconversion from NIR to UV was observed. The average decay times of green emission for TiO_2_:Er and TiO_2_:Er,Yb thin films were calculated based on time-resolved emission measurements.

## Abstract 2

TiO_2_:Er and TiO_2_:Er,Yb thin films deposited by magnetron sputtering showed upconversion from NIR to VIS with main emission in green (*λ*_em_ ≈ 525 nm). The higher content of activator and sensitizer resulted in increased upconversion and UV emission.

## Graphical abstract

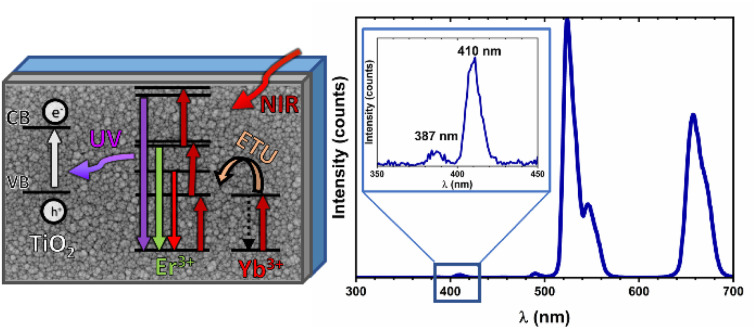

## Introduction

Lanthanide ions (Ln^3+^), along with transition metal ions and organic molecules, are well known to be luminescent centers in materials.^1,2^ The ladder-like structure of Ln^3+^ with abundant 4f energy levels enables multiple intraconfigurational transitions and allows not only light emission according to Stokes law but also the upconversion (UC) of two or more low-energy photons into a photon of a higher energy in the anti-Stokes upconversion process.^3^ Upconversion nanomaterials find applications in photonics, biomedicine, sensors, photocatalysis, photovoltaics, and photoelectrochemical cells.^4–10^

A typical upconversion material consists of emitting ions (activator), and optionally enhancing ions (sensitizer), embedded in a matrix (host).^8,11,12^ Er^3+^ ions are one of the extensively studied activator ions for upconversion from near-infrared (NIR) to visible (VIS), red and green, and ultraviolet (UV) regions (390–410 nm), which is due to an appropriate energy level structure.^11^ To enhance the absorption of infrared photons an Yb^3+^ sensitizer is added, due to its large absorption cross-section in NIR compared to other Ln^3+^ ions (9.11 × 10^−21^ cm^−2^ at 980 nm excitation).^13^ When ytterbium ions absorb infrared photons, electrons are excited from the ^2^F_7/2_ ground state to the higher energetic state. The excited state ^2^F_5/2_ of Yb^3+^ matches the f–f transitions in Ln^3+^ activators, for example it overlaps the ^4^I_11/2_ state of Er^3+^, thus excessive energy can be transferred to activator ions by the energy transfer mechanism.^14^ Choice of the host material is crucial due to its impact on the upconversion ability, emission intensity, and wavelength of emitted light.^5,11,12,15^ To achieve efficient luminescence, the host should be a low phonon material, which decreases the rate of non-radiative relaxation, with a good distribution of a high amount of activator ions to avoid cross-relaxation mechanisms. Usually, for inorganic upconversion materials fluorides (NaYF_4_), oxides (ZrO_2_, Y_2_O_3_, SiO_2_, TiO_2_), oxyhalides (GdOCl), oxysulfides (La_2_O_2_S), and phosphates (LuPO_4_, YPO_4_) are used as hosts.^5^

TiO_2_, in polymorphic forms of anatase, rutile and brookite, is an important semiconductor for photocatalysis and photoelectrochemical cells (PEC).^16–18^ In particular, its ability to utilize solar energy to split water into hydrogen and oxygen is applicable for green solar energy. However, the wide bandgap of TiO_2_ (*E*_g, rutile_ = 3.0 eV, *E*_g, anatase_ = 3.2 eV) limits light harvesting to UV, while VIS and NIR light are underutilized (sub-bandgap energy photons) and thus modification strategies such as microstructure modification, doping, formation of heterostructures, bandgap engineering, and enhancement by the upconversion process are investigated.^16,17^ The last mentioned strategy does not rely on the modification of TiO_2_ properties to better fit the solar spectrum, but rather on the change of the light distribution. Combining TiO_2_ with UC materials allows to absorb additional photons from the NIR range, then upconvert them to visible and UV that can excite TiO_2_.

To the best of our knowledge, the application of upconverters in photoelectrochemical cells for water splitting, contrary to well-developed upconverters for photovoltaic cells or photocatalysts, is still an unexploited research area.^17,19,20^ Moreover, most of the investigated UC materials are in the form of powders^21^ or bulk materials,^22^ like optical fibers, while upconversion thin film materials remain a challenge. To achieve upconversion and maintain good photoelectrochemical performance in case of a few hundred nanometers thick TiO_2_ film photoanode, it is important to find a balance between introducing a high amount of activator and sensitizer ions, their distribution in the host, and obtaining proper structure and microstructure of TiO_2_. In general, a higher activator concentration increases the possibility of upconversion, but after reaching a certain level, the activator ions are too close to each other and the UC efficiency is lowered due to the cross-relaxation mechanism. Meanwhile, the sensitizer ions need short distances between the ions, which allows for non-radiative energy transfer. Thus, another strategy is to keep the activator ion concentration at a low level, but with the addition of a sensitizer. In that way, the amount of activator ion can be reduced to a few mol%, while for the sensitizer it can reach a high concentration (≈20 mol%).^17^ Salhi and Deschanvres^23^ prepared crystallized TiO_2_ powders with 5 mol% Er and 10 mol% Yb *via* hydrothermal method. However, a high concentration of Ln^3+^ ions, due to significantly larger ionic radii than Ti^4+^, leads to the amorphization of TiO_2_. Pérez *et al.*^24^ investigated TiO_2_ films with Er^3+^ ions doping concentration up to 10 at%, for which diffraction peak of (1 0 1) anatase plane was still observable, but the amorphous TiO_2_ phase was dominant. Johannsen *et al.*^25^ showed that changes in host crystallinity in Er^3+^ doped TiO_2_ films deposited on Si (100) substrates significantly modified its luminescence properties. Moreover, red-to-green emission ratio relate to Er^3+^/Yb^3+^ concentration ratio as presented by Jung,^26^ while blue or UV light emission is observed only for UC materials with high efficiency due to low probability of the three or four photon UC processes.^9^

In our previous works, we investigated both the concepts of external^27^ and internal upconverter,^28,29^ and we demonstrated the possibility to use TiO_2_ thin film photoanodes modified with rare earth ions (Nd^3+^, Er^3+^, Yb^3+^) in photoelectrochemical cells.^27,29^ Thin film photoanodes deposited at 20% O_2_/(O_2_ + Ar) flow rate ratio exhibit the best photoelectrochemical properties (high value of photocurrent and rectangular shape of current density *versus* voltage curves).^27^ In our research on photoanodes with internal upconverter, we examined two strategies: introduction of high activator content (TiO_2_:Er (10 at%)) or incorporation of the low activator content with the addition of sensitizer (TiO_2_:Er,Yb (1 at%, 1 at%) and TiO_2_:Er,Yb (2 at%, 10 at%)). Recently, we described the photoelectrochemical properties of such photoanode materials.^29^ Here, we would like to focus on the investigation of TiO_2_ modified with Er and Yb thin films luminescence properties and the influence of the surrounding host on the lanthanide ions upconversion ability. We compare thin film upconverters with crystallized and amorphous host, which was achieved by changing the content of Er^3+^ and Yb^3+^ ions.

## Experimental

TiO_2_, TiO_2_:Er and TiO_2_:Er,Yb thin films were deposited in reactive radio frequency magnetron sputtering in an ultra-high vacuum system (PREVAC). The base vacuum in the process chamber, after heating up the substrates to 350 °C, was at the level of 10^−7^–10^−8^ mbar. During deposition, the argon and oxygen flow rates were set at 32 sccm and 8 sccm, respectively to keep 20% O_2_/(O_2_ + Ar) flow rate ratio, and the working pressure at the level of 6–7 × 10^−3^ mbar. Sputtering of 2′′ targets (Kurt Lesker Ltd) with diverse compositions, Ti, Ti/Er (90/10 at%), Ti/Er/Yb (98/1/1 at%), and Ti/Er/Yb (88/2/10 at%), was performed with a power density of 9–10 W cm^−2^. More details of process steps and parameters are described in our previous work.^28^ Thin films were deposited on various substrates: amorphous silica (Q) for investigation of the thin film structure, and to assess the impact of rare earth ions on the optical bandgap of the TiO_2_ host, silicon (Si) for observation of the thin film microstructure and further investigation of its structural properties, conducting indium tin oxide glass (ITO) to assess luminescence properties of upconversion photoanodes.

The average thickness of the deposited films was measured with a Rank Taylor Hobson Talystep stylus surface profilometer. The structure of the deposited thin films was determined by means of grazing incidence X-ray diffractometry (GIXRD) with a Philips X'Pert MPD diffractometer (*λ*_Cu_ = 0.154 nm). The diffractograms, with a subtracted background that originates from the silica substrate, were analysed according to data from the International Centre for Diffraction Data (ICDD) database (anatase 01-078-2486 and rutile 01-086-0147 cards). Scanning electron microscopy (SEM) imaging with energy dispersive spectroscopy (EDS) analysis was performed using Thermo Fischer Scientific Helios G4-PFIB-CXe microscope. For further structural investigation, X-ray absorption spectroscopy (XAS) measurements were conducted at SOLARIS National Synchrotron Radiation Centre in Kraków, Poland. XAS spectra were collected with synchrotron soft X-ray radiation at the PIRX beamline (Premiere InstRument for XAS, former PEEM/XAS). A more detailed description of the experimental procedure at the PIRX beamline is described in ref. 30. Measurements were carried out with 1 s per point acquisition time and energy steps of 0.05 eV for the pre-edge and white line range, and 0.1 eV over the range free from any sharp absorption peaks. Transmittance (*T*) and reflectance (*R*) spectra were measured with the UV-VIS-NIR Perkin Elmer double beam Lambda 19 spectrophotometer. Triax 180 Jobin Yvon grating monochromator with 450 W xenon lamp equipped with optical fiber and lens was used as a light source for photoelectrochemical experiments. Current *versus* time characteristics (*I*–*t*) were recorded under monochromatic illumination, changing the wavelength from 300 to 420 nm with a 10 nm step. 0.8 M Na_2_SO_4_ solution and a three electrode setup (working electrode – sample, auxiliary electrode – platinum foil covered with platinum black, reference electrode – Ag/AgCl in 3 M KCl) was used. The MTM Anko M161E electrochemical analyser served as a potentiostat for *I*–*t* measurements. Raman and photoluminescence spectra were collected with the WITec Alpha 300M+ spectrometer equipped with a 488 nm laser, the Zeiss LD EC 50× objective, and a CCD UV-NIR detector. Measurements in the 0–15 000 cm^−1^ wavenumber range were conducted in multiple scans, while each scan consisted of 2 seconds acquisition and 2 accumulations. Upconversion steady-state luminescence spectra and time-resolved emission measurements were performed in Horiba Fluorolog-QM-75-12C spectrofluorometer equipped with 700 mm double monochromator and photon counting PMT 920 IS detector with thermoelectric cooling, upon 980 nm semiconductor laser diode (LD) excitation.

## Results and discussion

Thin film samples are named according to the composition of the sputtering targets. The list of samples with target composition, temperature of substrate during deposition, deposition rate, and thickness measured with the profilometer can be found in Table 1.

**Table 1:** Sample names, deposition parameters and measured thin film thickness

Sample name	Target	Substrate heating temperature (°C)	Deposition rate (nm min^−1^)	Thickness (nm)
TiO_2_ crystallized	Ti	350	11.0	320
TiO_2_:Er (10 at%)	Ti/Er (90/10 at%)	350	7.7	230
TiO_2_:Er,Yb (1 at%, 1 at%)	Ti/Er/Yb (98/1/1 at%)	350	11.3	340
TiO_2_:Er,Yb (2 at%, 10 at%)	Ti/Er/Yb (88/2/10 at%)	350	8.2	270
TiO_2_ amorphous	Ti	No heating	7.3	220

**Fig. 1:**
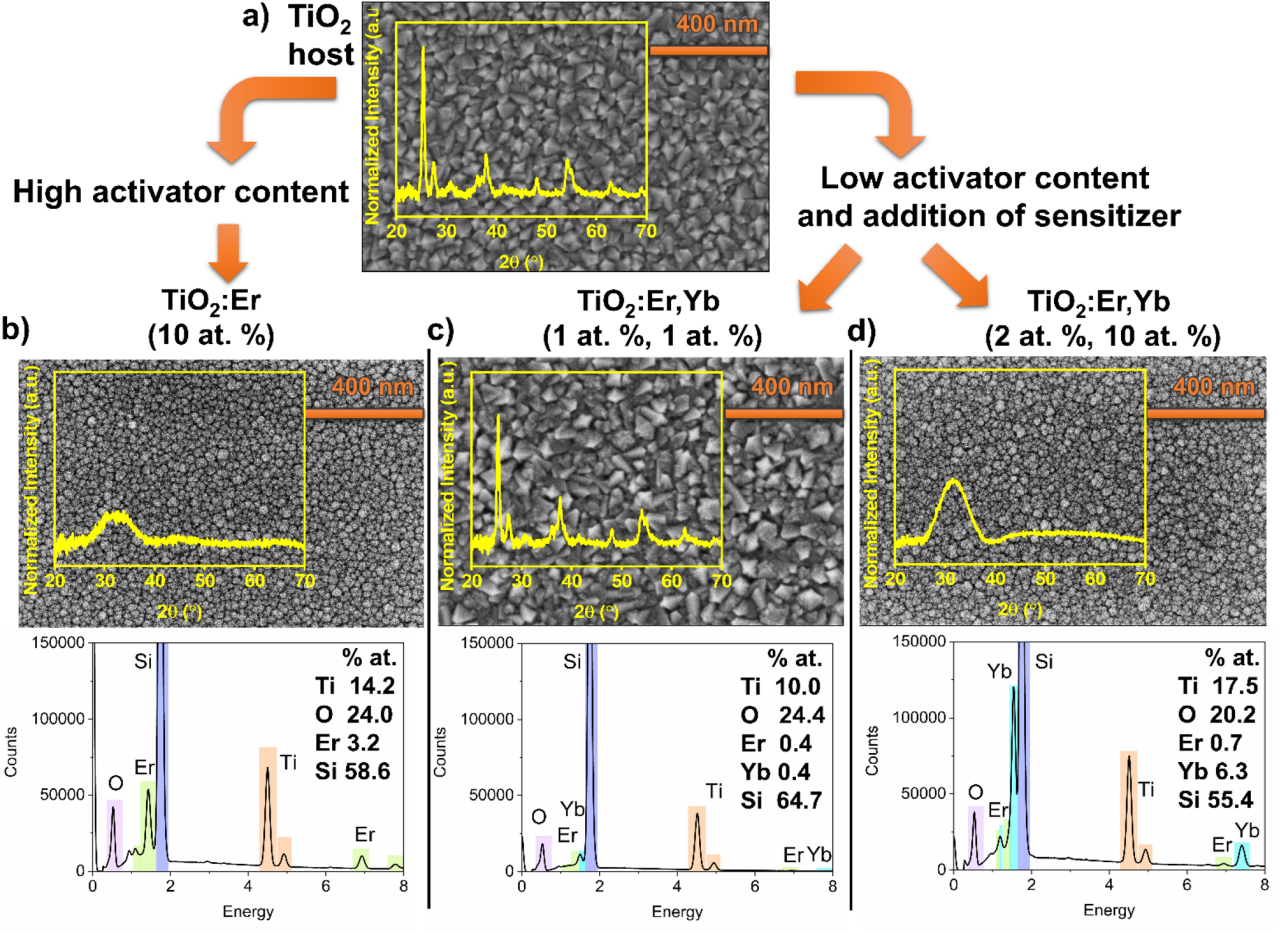
Diffractograms (GIXRD) and SEM images of (a) thin film crystallized TiO_2_ host, (b) modified thin film with high activator ions content TiO_2_:Er (10 at%), and (c and d) modified thin films with low activator content but with addition of sensitizer (c) TiO_2_:Er,Yb (1 at%, 1 at%), (d) TiO_2_:Er,Yb (2 at%, 10 at%). Additionally, EDS spectra with elemental analysis for modified thin films are presented.

To investigate the structure, microstructure and determine the elemental composition of thin films, GIXRD, SEM and EDS measurements were conducted (Fig. 1). Analysis of top-view SEM images and diffractograms showed that a high addition of Er and Yb ions caused amorphization of thin films, a decrease in average grain size, and a smooth surface compared to crystallized pure TiO_2_ or thin film with a low addition of Er and Yb. Crystallized TiO_2_ and TiO_2_:Er,Yb (1 at%, 1 at%) films have grains with pyramid-like tops with average grain size 57 and 52 nm, respectively. Anatase is a dominant phase, whereas rutile and a trace of brookite phase are also observed. For amorphous TiO_2_:Er (10 at%) the average grain size was 26 nm and for TiO_2_:Er,Yb (2 at%, 10 at%) 18 nm. It was reported that a high concentration of Er^3+^ seems to stabilize the TiO_2_ amorphous phase or hinder the crystallization.^31^ The ionic radii of Er^3+^ and Yb^3+^, 89 pm and 86 pm respectively, are significantly larger than Ti^4+^ ionic radius (68 pm). This mismatch has an impact on lattice distortion of TiO_2_.^20,32,33^ Therefore, substitution of Ti^4+^ in its lattice site is less probable than interstitial incorporation or segregation of Ln^3+^ ions at the grain boundaries.^20^ The EDS elemental analysis results (Fig. 1), considering the semi-quantitative character of this technique, correlate well with the composition of the targets used for the sputtering. In all EDS spectra, due to the beam penetration depth larger than film thickness, besides peaks from elements in the thin film (Ti, O, Er, and Yb), a high-intensity peak originating from the substrate (Si) is observed.

**Fig. 2:**
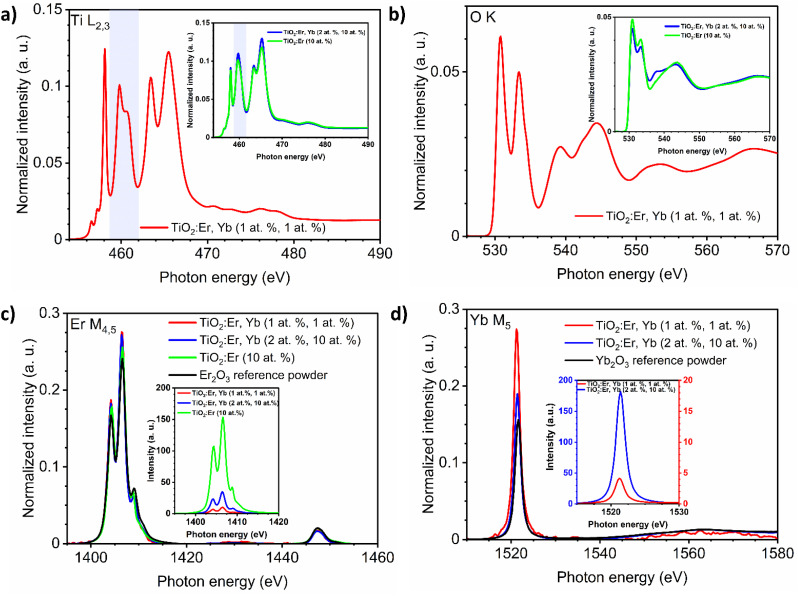
Normalized XAS spectra of: (a) Ti L_2,3_ and (b) O K edges of crystallized TiO_2_:Er,Yb thin films, in the insets amorphous TiO_2_:Er and TiO_2_:Er,Yb thin films. For the Ti L_2,3_ lines the L_3_-e_g_ peak is marked with a light blue rectangle. XAS spectra of: (c) Er M_4,5_ and (d) Yb M_5_ edges of TiO_2_:Er, TiO_2_:Er,Yb thin films, and reference lanthanide oxide powders (Er_2_O_3_, Yb_2_O_3_). In the insets, XAS spectra of the Er M_4,5_ and Yb M_5_ edges with intensity for the same measurement sensitivity are shown.

The presence of Er and Yb ions in films was also confirmed in the XAS measurements (Fig. 2). The absorption of synchrotron X-ray radiation in total electron yield (TEY) mode, as a very sensitive method, allowed to easily detect even very low addition of Er and Yb. The M_4,5_ Er and M_5_ Yb lines of thin films and reference oxide powders (Er_2_O_3_, Yb_2_O_3_) are presented in Fig. 2c and d. The area under the peak line in the XAS spectra collected for the same measurement sensitivity is dependent on the detected ions concentration. To compare the shape of the spectrum curves, it is necessary to perform a normalization procedure. The normalization procedure for the Ti L_2,3_ spectra was described in detail in our recent work,^30^ and here an analogous approach was applied for Ti L_2,3_, O K, Er M_4,5_ and Yb M_5_ spectra. The normalized Er M_4,5_ XAS spectra (Fig. 2c) have a similar shape, not affected by the phase composition of the samples. The same can be noticed for the normalized Yb M_5_ spectra (Fig. 2d). The Er M_5_ and M_4_ lines are related to 3d_5/2_ → 4f and 3d_3/2_ → 4f transitions with major absorption energies at 1404.9 and 1446.3 eV, respectively as reported in ref. 34. The collected spectra showed peak splitting of the Er M_5_ line into four maxima (1404, 1406, 1409, and 1411 eV), while for Er M_4_ one maximum at 1447 was observed. For Yb M_5_ only a single line was observed at 1521 eV, which can be attributed to 3d^10^4f_7/2_^13^ → 3d_5/2_^9^4f^14^ transition.^34^ Spectra in Fig. 2c and d insets are presented without normalization but for the same measurement sensitivity. Here, differences in the intensity of the signal are related to the amount of ions in the samples. The Ti L_2,3_ and O K lines are strongly affected by changes in phase composition (Fig. 2a and b). The Ti L_2,3_ spectrum with sharp peaks at the pre-edge, and the splitting of the L_3_-e_g_ peak at 460 eV indicated a well crystalized anatase and rutile phase, while for amorphous samples the L_3_-e_g_ peak splitting did not occur (inset in Fig. 2 a). For detailed analysis of Ti L_2,3_ lines, see ref. 30. The maxima of the O K lines at 531, 533, 539 and 544 eV can be assigned to 1s → t_2g_, 1s → e_g_, and 1s → a_1g_,t_1u_ transitions (see also ref. 35). The O K line for the crystallized sample (Fig. 2b) had better split t_2g_, e_g_, a_1g_, and t_1u_ peaks than the O K spectra of the amorphous films presented in the inset.

**Fig. 3:**
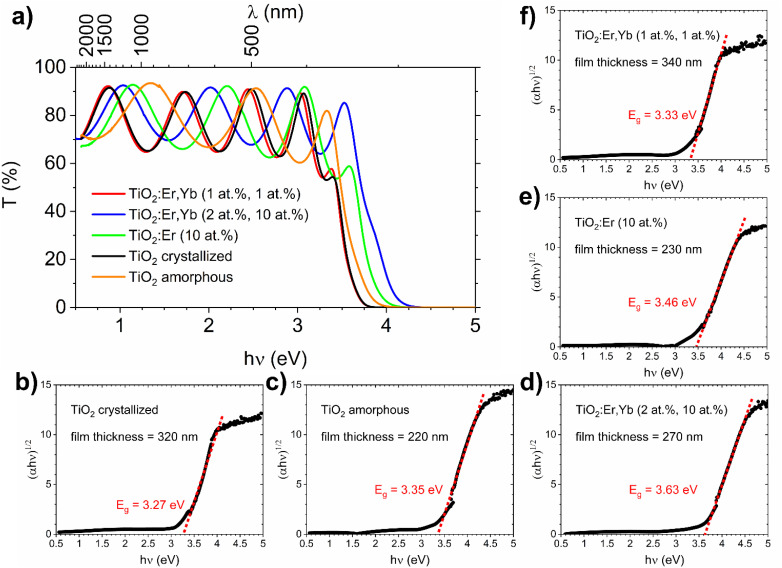
UV-VIS-NIR spectrophotometry results for TiO_2_, TiO_2_:Er, and TiO_2_:Er,Yb thin films. (a) Spectral dependence of the transmittance coefficient (*T*), (b–f) (*α*ℏ*ν*)^1/2^*vs.* ℏ*ν* dependence for optical bandgap *E*_g_ determination (Tauc method) with thin film thickness data from profilometer measurements. As a reference the amorphous TiO_2_ film results are provided.

The spectral dependence of transmittance coefficient (*T*) (Fig. 3a) showed oscillations of transmittance in the weak absorption region, which are due to the interference in thin films. The thin film thickness ranges from 220 to 340 nm as presented in Table 1 and Fig. 3b–f. As a reference, an additional TiO_2_ film was used – amorphous TiO_2_ film deposited at room temperature, which preparation and characterization were described in detail in our recent work.^36^ For both TiO_2_ and rare earth modified TiO_2_ amorphous thin films, a shift of the fundamental absorption edge to higher energies in comparison to crystallized films can be observed. The absorption coefficient (*α*) in the strong and weak absorption region was calculated using the “envelope method” from the transmittance and reflectance spectra taking into account the values of the thin film thickness measured with the profilometer. The optical bandgap *E*_g_ can be determined from the Tauc method based on eqn (1):

1 (*α*ℏ*ν*)^1/*γ*^ = *A*(ℏ*ν* − *E*_g_)

where *α* is the absorption coefficient, ℏ is the Planck constant, *ν* is the photon's frequency, factor *γ* is equal 2 for the indirect fundamental optical transition, *A* is a constant, *E*_g_ is the bandgap energy.^37^ The optical bandgap *E*_g_ values determined from the cut-off of the (*α*·ℏ*ν*)^1/2^*versus* ℏν dependence are presented in Fig. 3b–f. More details about this procedure can be found in our work on TiO_2_:Er thin films.^28^ Here, as references, crystallized (Fig. 3b) and amorphous (Fig. 3c) TiO_2_ thin films, with bandgap of 3.27 eV for nanosized anatase and 3.35 eV for amorphous TiO_2_, are shown. Rare earth modified films (Fig. 3d–f) have *E*_g_ = 3.33, 3.46, and 3.63 eV for TiO_2_:Er,Yb (1 at%, 1 at%), TiO_2_:Er (10 at%), and TiO_2_:Er,Yb (2 at%, 10 at%), respectively. The blue shift of TiO_2_:Er,Yb and TiO_2_:Er bandgap values could be associated with thin film amorphization or increased amount of Ln^3+^ ions. Determination of the bandgap energy from the Tauc method allows to factor in character of the material in the form of a thin film (dominant role of the thickness, and oscillations in transmittance and reflectance spectra) independently of the material functional properties.

The bandgap energy for photoanode materials can be also evaluated from photoelectrochemical methods.^38^ For a n-type film, if film thickness *L*_f_ is very small, the photocurrent *I*_ph_ and the absorption coefficient *α* relationship could be simplified to eqn (2):^39^

2 *I*_ph_ = *eJ*_0_*αL*_f_

where *e* is the electronic charge and *J*_0_ is incident light flux. Combining eqn (1) and (2), we achieve eqn (3):

3 (*I*_ph_ℏ*ν*)^1/*γ*^ = *B*(ℏ*ν* − *E*_g_)

where

4 *B* = *A*(*eJ*_0_*L*_f_)^1/*γ*^

**Fig. 4:**
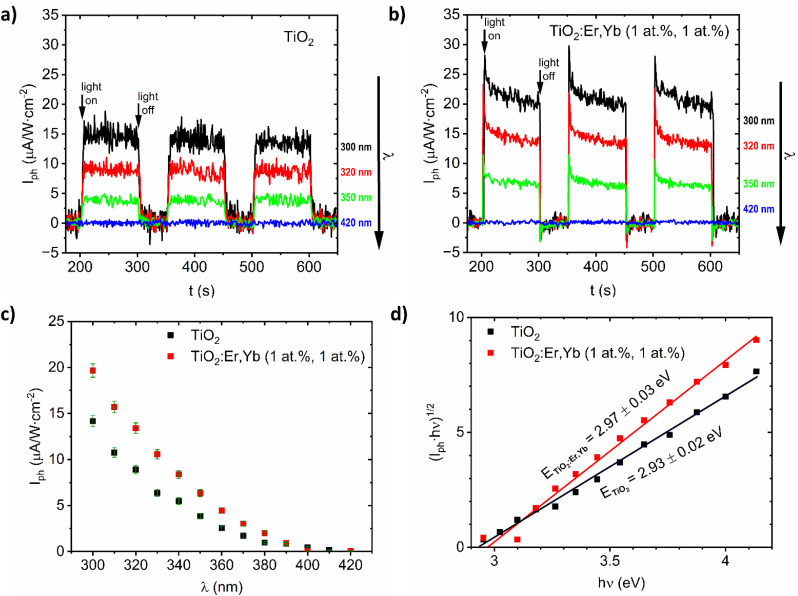
Selected characteristics of photocurrent *I*_ph_*versus* time (*t*) under monochromatic illumination (*λ* = 300, 320, 350, and 420 nm) for (a) crystallized TiO_2_ and (b) TiO_2_:Er,Yb (1 at%, 1 at%) thin films. (c) Spectral dependence of photocurrent *I*_ph_*vs. λ*, and (d) (*I*_ph_·ℏ*ν*)^1/2^*vs.* ℏ*ν* for photoelectrochemical bandgap *E* determination for crystallized TiO_2_ and TiO_2_:Er,Yb (1 at%, 1 at%) thin film photoanodes.

For TiO_2_ and TiO_2_:Er,Yb (1 at%, 1 at%) thin film photoanodes, which are samples with the best photoresponse (see results in our previous work^29^), this complementary method for bandgap determination based on photocurrent measurements was applied. From the photocurrent *I*_ph_*versus* time characteristics (Fig. 4a and b) the spectral dependence of photocurrent was determined and presented in Fig. 4c. The plots of (*I*_ph_·ℏ*ν*)^1/2^*versus* ℏ*ν* (Fig. 4d) allow to determine bandgap value *E* from the intersection of the line fitted to the experimental data with the photon energy ***h**ν* axis. For TiO_2_ and TiO_2_:Er,Yb thin films, bandgap value from photoelectrochemical measurements is 2.93 eV and 2.97 eV respectively. For both methods, TiO_2_ has slightly lower (0.04–0.05 eV) bandgap energy value than TiO_2_:Er,Yb (1 at%, 1 at%). The difference of about 0.3 eV between the bandgap energy evaluated from optical and photoelectrochemical measurements was also observed in other work.^40^

**Fig. 5:**
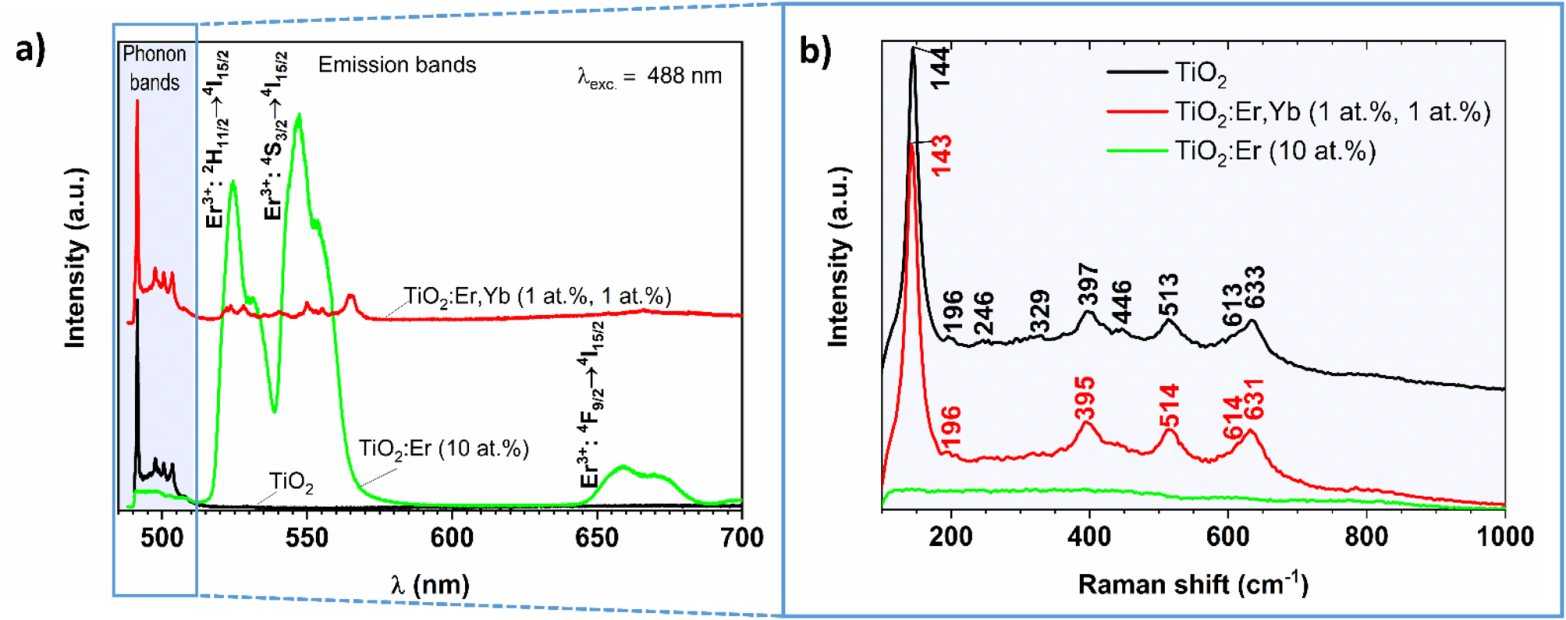
(a) Photoluminescence and (b) Raman spectra recorded for crystallized and amorphous rare earth modified TiO_2_ thin films and crystallized pure TiO_2_ reference thin film upon 488 nm laser excitation.

The photoluminescence spectra of crystallized and amorphous rare earth modified thin films are presented in Fig. 5a (the pure TiO_2_ thin film served as a reference). In this experiment, Raman and PL signals are collected at the same time, thus both TiO_2_ phonon bands (close to the 488 nm laser excitation line) and Er^3+^ emission bands (at higher wavelengths) can be observed. A similar setup for the PL measurement of Eu^3+^/TiO_2_ xerogels was described by Borlaf *et al.*^41^

In the phonon bands range for crystalized TiO_2_:Er,Yb (1 at%, 1 at%) thin film narrow lines at 490, 498, 501, and 504 nm can be observed, similarly to well-crystallized TiO_2_, while for amorphous TiO_2_:Er (10 at%) only increase in the noise level is observed. Detailed analysis of the 490–515 nm wavelength range is presented in Fig. 5b. The TiO_2_ phases were identified according to the following reference Raman modes: 144 (*E*_g_), 197 (*E*_g_), 400 (B_1g_), 516 (A_1g_ + B_1g_) and 640 cm^−1^ (*E*_g_) for anatase;^42^ 143 (B_1g_), 447 (*E*_g_), 612 (A_1g_), and 826 cm^−1^ (B_2g_) for rutile;^43^ 154 (A_1g_), 247 (A_1g_), 320 (B_1g_), and 329 (B_2g_) cm^−1^ for brookite.^44^ The obtained Raman shift values compared with the reference data confirm that the crystallized samples are a mixture of anatase, rutile and brookite (Table 2). The recorded PL spectra (Fig. 5a) allowed for investigation of the luminescence properties and analysis of the optical quality of the thin film. The sample with high Er^3+^ activator content showed high emission in green, peaks with the main maximum at 524 and 547 nm, which corresponds to ^2^H_11/2_ → ^4^I_15/2_, and ^4^S_3/2_ → ^4^I_15/2_ transitions in Er^3+^ respectively, and weak emission in red with the maximum at 659 nm (^4^F_9/2_ → ^4^I_15/2_). Pure TiO_2_ does not exhibit additional emission peaks in the visible light range. The results of PL proved the lack of clustering within Er^3+^ ions and the possibility of considering thin films in energy conversion systems.

**Table 2:** Raman shift of TiO_2_ and TiO_2_:Er,Yb crystallized films upon 488 nm laser excitation with comparison to the literature data provided for anatase, rutile, brookite; upconversion emission maxima of TiO_2_:Er and TiO_2_:Er,Yb films with assigned transitions (excitation wavelength 980 nm)

Raman shift (cm^−1^) upon 488 nm laser excitation					Upconversion emission wavelength *λ* (nm) upon 980 nm laser excitation			
TiO_2_ crystallized	TiO_2_:Er,Yb (1 at%, 1 at%)	Anatase^42^	Rutile^43^	Brookite^44^	TiO_2_:Er,Yb (1 at%, 1 at%)	TiO_2_:Er,Yb (2 at%, 10 at%)	TiO_2_:Er (10 at%)	Transitions^24,46^
144	143	144 (*E*_g_)	143 (B_1g_)	154 (A_1g_)		387		^4^G_11/2_ → ^4^I_15/2_
196		197 (*E*_g_)				410		^2^H_9/2_ → ^4^I_15/2_
246				247 (A_1g_)	524	524	523	^2^H_11/2_ → ^4^I_15/2_
329				320 (B_1g_), 329 (B_2g_)	538			^2^H_11/2_ → ^4^I_15/2_
397	395	400 (B_1g_)			551	546	545	^4^S_3/2_ → ^4^I_15/2_
446			447 (*E*_g_)		562			^4^S_3/2_ → ^4^I_15/2_
513	514	516 (A_1g_ + B_1g_)			660	658	659	^4^F_9/2_ → ^4^I_15/2_
613	614		612 (A_1g_)					
633	631	640 (*E*_g_)						

**Fig. 6:**
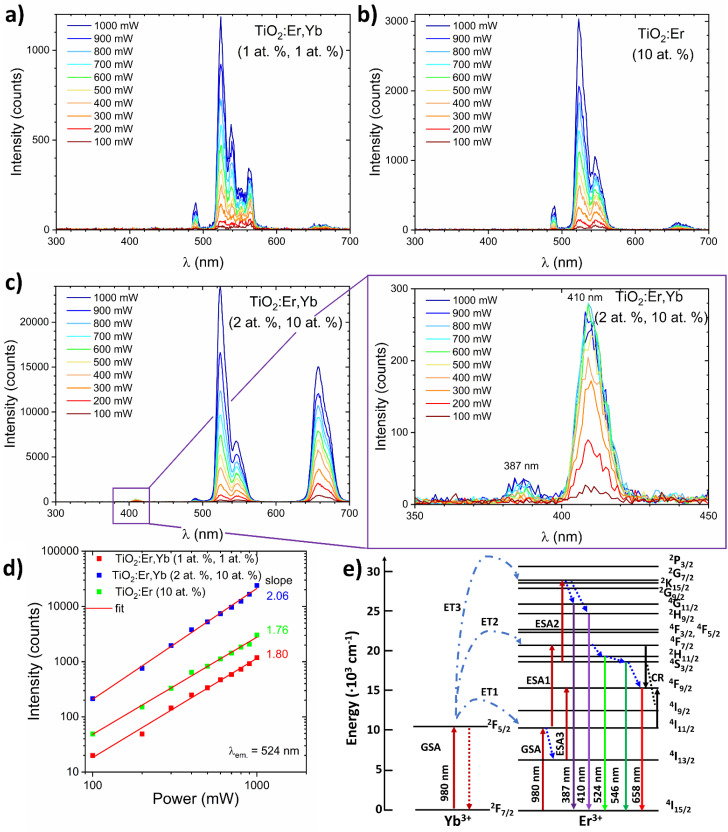
(a–c) Dependence of the upconversion spectrum of TiO_2_ and TiO_2_:Er,Yb thin films on the excitation power of the 980 nm laser diode. (d) Logarithmic plot of the green emission intensities of Er-, and Yb-modified thin films as a function of the laser pump power. (e) Upconversion transitions in a Yb^3+^/Er^3+^ couple upon 980 nm excitation. The dash and dot lines symbolize energy transfer (ET), the dote lines represent relaxation, the full lines indicate ground state absorption (GSA), excited state absorption (ESA), cross-relaxation transitions (CR) and radiative decay.

To analyse the upconversion properties of the photoanodes (rare earth modified TiO_2_ thin films on the ITO substrate), emission spectra were collected under the 980 nm laser diode excitation with laser pump power adjusted within the 100–1000 mW range (Fig. 6a–c). Measurements in a function of the pumping power confirmed the non-linear character of luminescence (Fig. 6d). All samples exhibit the main emission peak in the green wavelength range (*λ* ≈ 524 nm). TiO_2_:Er,Yb (2 at%, 10 at%) showed one order of magnitude higher green emission intensity than TiO_2_:Er,Yb (1 at%, 1 at%) and TiO_2_:Er (10 at%). The emission spectrum of the crystallized sample, similarly to the XAS spectra, has more split peaks, as presented in Table 2. The peak located at 490 nm wavelength in the UC spectra has origin from the second harmonic of the laser generated in the samples. On the basis of TiO_2_:Er and TiO_2_:Er,Yb spectra (Fig. 6a–c), it was observed that the addition of a high concentration of Yb ions significantly increases intensity of the upconversion emission. It occurs due to the following probable mechanisms^45^ (schematic diagram of UC processes shown in Fig. 6e):

(1) Due to two subsequent transfers (ET_1_, ET_2_) from ytterbium ion (^2^F_5/2_) the ^4^F_7/2_ Er^3+^ level is directly excited. Non-radiative relaxations from ^4^F_7/2_ Er^3+^ level populate the following ^2^H_11/2_, ^2^H_11/2_ and ^4^F_9/2_ levels. Then, radiative emissions at 524 nm, 546 nm and 658 nm occur as a result of transitions from ^2^H_11/2_, ^2^H_11/2_ and ^4^F_9/2_ levels to the ground state ^4^I_15/2_ respectively.

(2) Nonradiative transition ^4^I_11/2_ → ^4^I_13/2_ may promote population (LD and ET from Yb is possible) of the ^4^F_9/2_ level (ESA3) from which radiative relaxation ^4^F_9/2_ → ^4^I_15/2_ enhances emission in red.

(3) In result of high concentration, two adjacent Er^3+^ ions undergo a cross-relaxation according to ^4^F_7/2_ + ^4^I_11/2_ → ^4^F_9/2_ + ^4^F_9/2_ scheme (CR).

(4) In the presence of high concentration of Er, Yb (2 at%, 10 at%) the third Yb photon (ET3) may excite ^4^S_3/2_ Er ions to the ^2^G_9/2_ level. Then, after non-radiative relaxation to the ^4^G_11/2_ and ^2^H_9/2_ levels, emissions at 384 and 410 nm take place after the transition to the ground state.

In case of higher than NaYF_4_ phonon host like TiO_2_, both multi-phonon and cross-relaxation mechanisms are promoted. It is consistent with the results of log-log plots (*λ*_em_ = 524 nm, Fig. 6d) where the ET3 is possible only for the sample doped with 10 at% of Yb where slope exceeded 2.0 value. Increasing the total content of Ln^3+^ ions changes luminous centers surroundings which is indicated by the GIXRD and XAS results. Moreover, the distances between Er^3+^ and Yb^3+^ ions are reduced as the Ln^3+^ content increases, which increases UC probability and CR leading to enhanced UV-red emission.

Jung^26^ reported that the main UC emission color of TiO_2_:Er^3+^/Yb^3+^ phosphors is rather controlled by the Yb^3+^ than the concentration of Er^3+^ – with increasing Yb^3+^ ions concentration the red-to-green ratio increased significantly. Fig. 6d shows a logarithmic plot of the green emission intensities of rare earth modified thin films as a function of the laser pump power. The slope values close to 2 (2.06, 1.76, and 1.80 mW) indicate that this level is populated in two-photon process, which is in accordance with Er^3+^ energy diagram.^9^ For TiO_2_:Er,Yb (2 at%, 10 at%) thin film, UV (387 nm) and violet (410 nm) upconversion emission was observed (Fig. 6c inset), confirming the high efficiency of this UC material. Moreover, achieving upconversion from IR to UV is very attractive to enhance performance of TiO_2_ based photoanodes. Based on energy diagrams in ref. 46–48 possible mechanism of three-photon upconversion in the Yb^3+^ and Er^3+^ couple is presented in Fig. 6e and can be described as follows. After two-photon excitation from the ^4^I_15/2_ ground state to the ^4^F_7/2_ excited state, multi-phonon relaxation to ^4^F_9/2_ occurs. Absorption of third 980 nm photon allows excitation to ^2^G_7/2_, then relaxation to the ^2^H_9/2_ or ^4^G_11/2_ energy level takes place. During the ^2^H_9/2_, ^4^G_11/2_ → ^4^I_15/2_ transition 410 and 387 nm photons are emitted.

**Fig. 7:**
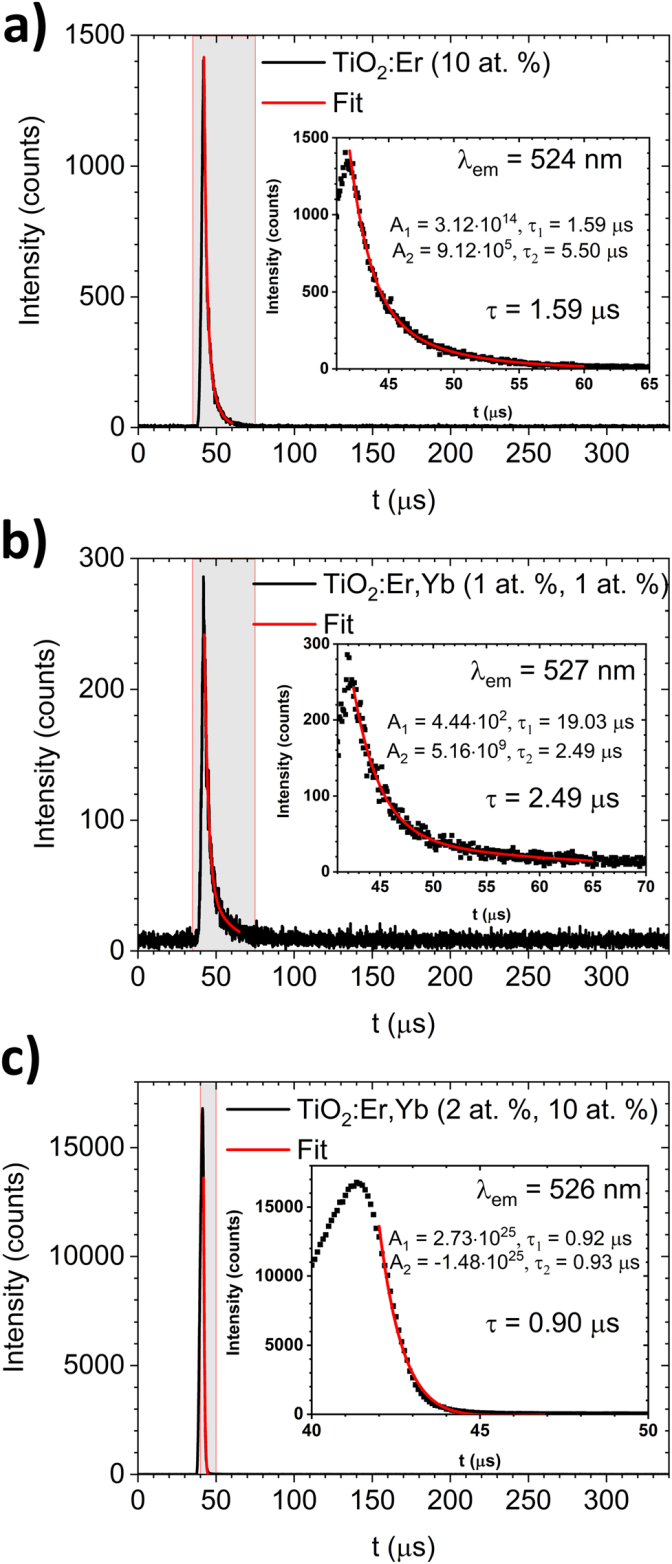
The upconversion luminescence decay for green emission (*λ*_em_ ≈ 525 nm) of (a) TiO_2_:Er and (b and c) TiO_2_:Er,Yb thin films upon 980 nm laser diode excitation. In the insets, results of the decay curve fitting and values of average decay time are shown.

The luminescence decay profiles (Fig. 7) were measured under 980 nm excitation. The decay curves for green emission ^2^H_11/2_ → ^4^I_15/2_ (*λ*_em_ ≈ 525 nm) were fitted according to eqn (5):

5 *I*(*t*) = *A*_1_ exp(−*t*/*τ*_1_) + *A*_2_ exp(−*t*/*τ*_2_)

where *I* is intensity, *t* – time, *τ*_1_, *τ*_2_ are decay times, *A*_1_, *A*_2_ are fitting constants. The values of the average decay time *τ* were calculated as the weighted average:^49^6
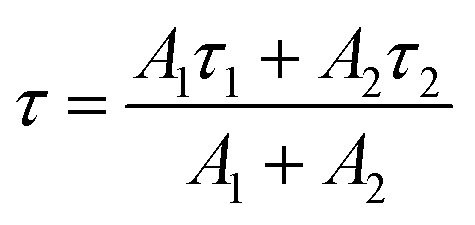


For TiO_2_:Er and TiO_2_:Er,Yb thin films, the average decay times *τ* of green emission were several μs (0.90–2.49 μs) as shown in Fig. 7. Lakhotiya *et al.*^49^ reported TiO_2_:Er thin films sputtered and annealed at 350 °C, which upon 800 nm laser excitation have a decay time of infrared emission (^4^I_11/2_ → ^4^I_15/2_ transition) around 10 μs. Zhanci *et al.*^50^ showed that the measured lifetime of the erbium ion ^4^S_3/2_ and ^2^H_11/2_ states in oxyfluoride tellurite glass at 303 K is approximately 20 μs.

## Conclusions

The thin film with small addition of activator and sensitizer TiO_2_:Er,Yb (1 at%, 1 at%) was a well-crystallized mixture of anatase, rutile and brookite, similarly to pure TiO_2_, while the high addition of activator or sensitizer ions in TiO_2_:Er (10 at%) and TiO_2_:Er,Yb (2 at%, 10 at%) thin films led to amorphization as it was evaluated from GIXRD and XAS measurements. EDS and XAS measurements confirmed that erbium (activator) and ytterbium (sensitizer) ions were introduced into the TiO_2_ host. The TiO_2_:Er and TiO_2_:Er,Yb thin films deposited in the magnetron sputtering process showed upconversion from NIR to VIS with main emission in green (*λ*_em_ ≈ 525 nm). Both TiO_2_:Er,Yb (1 at%, 1 at%) and TiO_2_:Er (10 at%) showed weak emission in red (*λ*_em_ ≈ 660 nm), while for TiO_2_:Er,Yb (2 at%, 10 at%) thin film increased red emission and even upconversion from NIR to UV was observed. Thus, it can be concluded that the higher content of activator and sensitizer allowed for more efficient upconversion, and the thin film ability to upconvert NIR photons was not affected negatively by the amorphization. Both UC spectra and XAS spectra (Ti L_2,3_ and O K edges) are more split for crystallized samples than for amorphous ones. The emission spectrum depends not only on the host type but also on the activator and sensitizer ions surroundings. As shown in XAS spectra, samples with high erbium (10 at%) and ytterbium (10 at%) content have different surroundings than sample with low Er and Yb addition. It has an impact on the average decay times of green emission. The average decay time of a several μs (typical for the TiO_2_ host) decreases with increasing total Ln^3+^ content from 2.49, through 1.59 to 0.90 μs. Further optimalization of thin film composition, structure, microstructure, and optical properties to achieve high upconversion from NIR to UV and to maintain high photoelectrochemical performance is crucial for the development of efficient upconverting TiO_2_ photoanodes for photoelectrochemical water splitting.

## Conflicts of interest

There are no conflicts to declare.

## Supplementary Material
